# Editorial: Bone and metabolic activities

**DOI:** 10.3389/fdmed.2023.1244033

**Published:** 2023-07-26

**Authors:** T. E. Van Dyke, N. E. Hatch, K. Joshipura, N. E. Lane, M. Murshed, F. Nociti, B. Olsen, M. J. Somerman

**Affiliations:** ^1^ Department of Applied Oral Sciences, Forsyth Institute, Cambridge, MA, United States; ^2^ Faculty of Medicine, Harvard University, Boston, MA, United States; ^3^ Department of Orthodontics and Pediatric Dentistry, School of Dentistry, University of Michigan, Ann Arbor, MI, United States; ^4^ School of Public Health, Ahmedabad University, Ahmedabad, India; ^5^ Department of Medicine, University of California at Davis School of Medicine, Sacramento, CA, United States; ^6^ Division of Endocrinology and Metabolism, McGill, Montreal, QC, Canada; ^7^ American Dental Association Science and Research Institute, ADA, Gaithersburg, MD, United States; ^8^ National Institute of Dental Craniofacial Research, National Institutes of Health, Bethesda, MD, United States

**Keywords:** bone, systems biology, endocrine, osteology, transgenic mice, metabolism

**Editorial on the Research Topic** Bone and metabolic activities

## 1. Introduction

Substantial progress has been made in defining genes and proteins involved in the development, maintenance and regeneration of teeth and bones. This knowledge has improved strategies for both the diagnosis and treatment of mineralized tissue diseases. Existing data provide credence for these genes/proteins to have roles beyond those attributed to mineralized tissues, including acting as endocrine factors, and altering metabolic activity at distant sites (1). However, there remains substantial uncertainty as to what extent bone itself functioning as an endocrine organ, and/or factors secreted by bone, modulate metabolic activity. This Research Topic was developed to advance our understanding of the effect of hard tissues on metabolic activity, to provide information of value in considering clinical strategies to prevent and treat disorders of mineralized tissue and metabolic activity. This Research Topic is comprised of original research articles, reviews, mini-reviews and perspectives that support the influence of mineralized tissues on metabolism.

## 2. Original articles (4)

There is strong evidence supporting bidirectional effects of chronic periodontal disease and diabetes mellitus on overall health status (2). Further, diabetes is associated with higher risk of long bone and jaw fractures. Two original articles focus on diabetes. In the manuscript by Heikkilä et al., in a ten year follow up study using an impressive cohort of 68,273 compromised patients, they provide additional data in support of an association between chronic oral diseases and diabetes. The inability of individuals with diabetes to regulate insulin levels and related glucose abnormalities is known to compromise the health of numerous tissues of the body including teeth and bones, plus downstream effects on whole body metabolic activity.

In the article by Alharbi and Graves, they induced diabetes in mice with a targeted FOX01 deletion in chondrocytes vs. controls. The results emphasize the important role of FOX01 in modulating a variety of factors associated with a diabetic profile. In another study by Zebrowitz et al., using a mouse model of periodontal disease, periodontally diseased teeth were treated with a timed-release formulation of a small molecule inhibitor of prolylhydroxylases (PHDi; 1,4-DPCA), previously shown to induce epimorphic regeneration of soft tissue in non-regenerating mice. PHDi induces high expression of HIF-1α, a target gene for 1,4-DPCA, and is able to shift the cellular metabolic state from oxidative phosphorylation to aerobic glycolysis, an energetic state used by mesenchymal stem cells and embryonic tissue. The authors showed evidence of metabolic reprograming by increased expression of HIF-1α, as well as metabolic genes *Glut-1, Gapdh, Pdk1, Pgk1* and *Ldh-a* in periodontal tissues.

The data provided in the manuscript by Nagasaki et al., using a mouse model where the arginine-glycine-aspartic acid (RGD) region of bone sialoprotein is replaced by the nonfunctional sequence of lysine, alanine, glutamic acid (KAE knockin), reported that KAEKI mice vs. control mice developed mild obesity, an increase in body weight, adipocyte hypertrophy in white epididymal fat and interscapular brown fat, dyslipidemia and hyperleptinemia but no significant changes in glucose metabolism suggesting that the RGD region of BSP affects energy metabolism by regulating food intake.

### 2.1. Reviews (1)

In a comprehensive review by Franceschi et al. on the role of discoidin domain receptors in controlling bone development, regeneration and metabolism, they provide evidence that in addition to the know interactions of specific β1 integrins and collagen receptors in bone, a second, more primordial collagen receptor family, the discoidin domain receptors also play a critical role in mineralized tissue development as well as related functions in abnormal bone formation, regeneration and metabolism.

### 2.2. Mini Reviews (2)

Two mini reviews center on periodontal tissues, including one by Tazawa et al. on the role of IL-1 signaling in development of apical periodontitis (AP) and the other by Abdalla and Van Dyke on the effect of the soluble expoxide hydrolase (eHS) cascade on periodontal tissues. Tazawa et al. review evidence in support of previous data linking increased alkaline phosphatase enzyme (AP) with obesity and specifically the role of IL-1RA in regulating IL-1 signaling and modulating apical lesion progression in obesity. Abdalla and Van Dyke discuss the mechanism by which eHS inhibitors enhance the production of pro-resolving mediators to affect periodontitis, and further that such knowledge may inspire novel approaches to prevent and treat periodontal diseases.

### 2.3. Perspectives (2)

Nagaski et al. discuss the mounting evidence of the role mineralized tissues and associated factors in controlling whole-body metabolism, including metabolic disorders such as diabetes and obesity, while Fraser and Ganesan provide new insights as to the significance of interactions between oral and gut microbiome, and alveolar bone and associated metabolites in health and disease. They highlight that the advances in metabolomics, transcriptomic and metagenomic technologies should assist in identifying novel metabolites affecting the health of mineralized tissues.

Together, this diverse set of articles invites the reader to consider their own research areas and to rethink, if not already reimagining, the potentially significant role of bones and teeth in influencing metabolic activity in health and in diseased states. We welcome additional articles in this field for publication in FMED or FOH.
